# Address to Graduates of the Grady Hospital Training-School for Nurses, May 13, 1900

**Published:** 1900-08

**Authors:** Virgil O. Hardon

**Affiliations:** Atlanta, Ga.


### ADDRESS TO GRADUATES OF THE GRADY HOSPITAL TRAINING-SCHOOL FOR NURSES, MAY 13, 1900.

By VIRGIL O. HARDON, M.D.,

ATLANTA, GA.

It is a trite observation, that to those of us who can look backward through the vista of a quarter or a half century the advances which have been made during that period in all departments of science and of art seem little short of the miraculous. We have but to look about us to find incontrovertible evidences of the truth of this statement. Our every-day life is filled with them, and constant association alone has made them seem commonplace to us. In no department of human endeavor have greater advances been witnessed than in the care of the sick. I do not refer to the improvements in medical and surgical practice, wonderful as they have been, but to the branch of treatment which is comprised under the title of nursing. Above fifty years ago that great analyst of human character, Charles Dickens, gave a description of what may be regarded as a typical nurse of that day. Of Sairey Gamp he says: "She was a fat old woman, this Mrs. Gamp, with a husky voice and a moist eye, which she had a remarkable power of turning up and showing only the white of it. Having very little neck it cost her some trouble to look over herself, if one may say so, at those to whom she talked. She wore a very rusty black gown, rather the worse for snuff, and a shawl and bonnet to correspond. In these dilapidated articles of dress she had, on principle, arrayed herself time out of mind, on such occasions as the present. The face of Mrs. Gamp, the nose in particular, was somewhat red and swollen, and it was difficult to enjoy her society without becoming conscious of the smell of spirits. Like most persons who have attained to great eminence in their profession, she took to hers very kindly—and she went to a lying-in or a laying-out with equal zest and relish."

To those of you who have always lived in the South where the kindness and tenderness of the negro "mammy" atoned to a large extent for her lack of skill, it may seem that this description of the novelist is but a figment of a fertile brain. But medical men who have practiced north of Mason and Dixon's line will recognize the picture as a true one. We have seen Sairey Gamp, and, unfortunately, her race is even yet not quite extinct. Like the dodo and the bison, a few rare specimens still remain to link us to a past age. If I wish to emphasize the difference between the so-called nurse of the Sairey Gamp epoch and the trained nurse of to-day, I need only quote the words of the immortal bard and say: "Look on that picture, and then on this."

I propose in this address, young ladies, to touch briefly upon some of the attributes which I conceive to be necessary to the making of a good nurse. Your training has comprised an infinitude of details, each one of which is important and essential. But there is a possibility that in the cultivation of your knowledge of details you may have lost sight of the great underlying principles upon which they rest. The qualifications of a good nurse are, to my mind, comprised under three heads.

The first of these is technical skill. The vocation of nursing is a profession, because it involves a thorough knowledge of everything which relates to it. It is also an art, because it involves the ability to apply that knowledge to practical uses. It is the skill of the trained nurse which distinguishes her from the amateur. It is for the purpose of acquiring this skill that you have spent three of the best years of your life in this school. Trained nursing implies a perfection in details which no amount of natural aptitude can impart. Just as the so-called natural musician must spend many weary hours in practicing scales and octaves before he can become a virtuoso, so the nurse, however much of natural aptitude she may possess, must perfect herself in all the minutest details of her art before she can claim to be trained. And not only must she know methods, but she must know the *best* methods. Any one can make up a bed, but you all know that to perform this seemingly humble task in the best way requires practice and a knowledge of details which is not possessed by the average woman. Any one can give a thirsty patient a glass of water, but you have all learned that there is a right way and a wrong way in which to do it. The difference between deftness and clumsiness is nowhere more apparent than in the performance of this trivial duty. No one but he who has experienced it knows the luxury of a sponge bath when administered by the skilled hands of the trained nurse, as compared with the bungling, even though well intended, efforts of the amateur. And so I might illustrate this idea by citing all the minutiæ of which your training has been made up. The best way to learn to do a thing well is by doing it over and over again. And that is the way in which you have been taught. Not by theory, but by actual bedside practice, day after day, under competent instruction. Upon the foundation thus laid should be reared an ever-increasing skill, to which the duration of your life alone can set a limit.

But it is not enough simply to know the details of your art, lest you might bring upon your heads the condemnation of the lost spirits of Dante's Inferno: "Ye knew your duty and ye did it not." To the trained nurse the proper performance of duty must become a matter of conscience. An aseptic conscience is as necessary in your vocation as aseptic hands. Though you may possess all the skill that comes with years of training and of experience, yet without thorough and absolute conscientiousness your knowledge will be but as "sounding brass and tinkling cymbals." Your work is of a kind which must be done with absolute precision and regularity, without regard to your own personal feelings and preferences. There will be times when the brow will throb with pain, when the heart may seem near to bursting with some sorrow, even when life itself seems for the time to be an intolerable burden. Yet all these things must be left outside the door of the sick-room and not obtruded upon the attention of your patient to add to her sufferings. The trained nurse should be the faithful coadjutor of the physician. To her he looks for the successful carrying out of his treatment of his patient. To her his directions should be as the laws of the Medes and Persians. Nothing but an absolute conscientiousness on your part can give to the physician this confidence. Clean hands and a clean professional conscience should go side by side, since both are equally essential to the making of the ideal nurse.

There is another element which should enter into the formation of your character, but which perhaps has not been so often brought to your attention, since it is not of a kind which can be acquired by didactic teaching—but to my mind it is of transcendent importance to your success—and that is, an unlimited amount of human sympathy. Not the sympathy which shows itself in the suffused eye, in the blanched cheek, in the quivering lip, or in the trembling hand. That is but physical weakness, and as such should be overcome. But I refer to the sympathy which wells up from the heart, and which can make the ministrations of the trained nurse as tender and as soothing as the touch of a mother's hand upon the brow of her sick babe. Such sympathy is positively curative in its effect. Just as the face may become suffused with a blush under the influence of an emotion acting through the nervous system, just so every organ of the body may be stimulated to a renewed vitality under the influence of a kind look, a sympathetic word or a tender touch. This is no new or fanciful theory. If you search the Scriptures you will find that more that twenty-five centuries ago Solomon, the wise man, recognized this truth when he said "A merry heart doeth good like a medicine." Such sympathy, therefore, should be a part of your equipment for your work, and should be cultivated in the same way that you have trained your minds and your consciences. Why is it that the vocation of nursing the sick has been given over almost entirely to your sex? It is because nature has endowed you with a capacity for such sympathy far in excess of that which she has bestowed upon us. The same inborn desire to care for and cherish the weak and helpless which shows itself in the maternal instinct that is awakened in every mother's heart at the first cry of her child, that is the quality which distinguishes you from the more logical but less emotional sterner sex and preeminently fits you for the office of a trained nurse.

> "Oh, woman, in our hours of ease
> Uncertain, coy and hard to please,
> When pain and anguish wring the brow
> A ministering angel thou."

Skill, conscientiousness, sympathy—the trinity of jewels which should adorn the tiara of the trained nurse. If either of these be lacking the crown of your training will be incomplete.

And now, young ladies, I bid you Godspeed as you go forth to the vocation which you have chosen. May the years which stretch out before you bring a full realization of all the hopes and aspirations which have thrilled your hearts during the period of your preparation. May the lessons which you have learned in this school be enriched by an ever-broadening experience as the years go by. May the Great Physician himself smile upon your labors and make you a blessing to suffering humanity in your day and generation.

